# Glycosylation in the tumor immune response: the bitter side of sweetness: Glycosylation in the tumor immune response

**DOI:** 10.3724/abbs.2024107

**Published:** 2024-06-28

**Authors:** Yuting Cao, Wen Yi, Qiang Zhu

**Affiliations:** Department of Biochemistry College of Life Sciences Zhejiang University Hangzhou 310058 China

**Keywords:** glycosylation, immune response, endogenous lectins, immune checkpoint, extracellular matrix, immunotherapy

## Abstract

Glycosylation is the most structurally diverse form of post-translational modification (PTM) of proteins that affects a myriad of cellular processes. As a pivotal regulator of protein homeostasis, glycosylation notably impacts the function of proteins, spanning from protein localization and stability to protein-protein interactions. Aberrant glycosylation is a hallmark of cancer, and extensive studies have revealed the multifaceted roles of glycosylation in tumor growth, migration, invasion and immune escape Over the past decade, glycosylation has emerged as an immune regulator in the tumor microenvironment (TME). Here, we summarize the intricate interplay between glycosylation and the immune system documented in recent literature, which orchestrates the regulation of the tumor immune response through endogenous lectins, immune checkpoints and the extracellular matrix (ECM) in the TME. In addition, we discuss the latest progress in glycan-based cancer immunotherapy. This review provides a basic understanding of glycosylation in the tumor immune response and a theoretical framework for tumor immunotherapy.

## Introduction

Glycosylation is a common form of protein and lipid modification by the covalent attachment of saccharides to proteins and lipids. The major types of glycosylation in mammals include N-glycosylation, O-glycosylation, O-GlcNAcylation, GPI-anchored glycoproteins, and glycosaminoglycans [1]. N-glycosylation, in which N-glycans are attached to asparagine within the conserved N-X-S/T sequon (where X represents any amino acid excluding proline) in peptides, frequently occurs in membrane proteins [1]. All N-glycans possess a core pentasaccharide; however, the diversity of monosaccharides and the complexity of linking patterns result in different types of N-glycans, which are divided into high-mannose, hybrid, and complex types [2]. In addition to N-glycosylation, O-glycosylation and O-GlcNAcylation occur at serine/threonine (S/T) residues [1]. O-glycosylation often refers to the glycosylation of glycans initiated by N-acetylgalactosamine (GalNAc). Biosynthesis of N-glycans is initiated in the endoplasmic reticulum (ER) and further elongated in the Golgi apparatus, while the entire synthesis of O-glycans occurs in the Golgi apparatus [3]. In contrast to N-glycans and O-glycans, O-GlcNAcylation is a unique monosaccharide β-N-acetylglucosamine (GlcNAc) modification that predominantly occurs in intracellular proteins and is mediated solely by O-GlcNAc transferase (OGT) and the O-GlcNAcase (OGA) [4].

Glycosylation functions as a critical regulator of multiple physiopathological processes, including signal transduction and communication, invasion, cell–matrix interactions and immune modulation [5]. Therefore, deregulation of glycosylation is closely associated with tumor development and progression. Accumulating evidence highlights that aberrant glycosylation plays a pivotal role in tumor immunity through influencing the interaction of glycosylation receptors, lectins and ligands on the cell surfaces of tumor cells and immune cells. For example, tumor cells secrete galectins to promote regulatory T cells (Tregs) and T-cell exhaustion by impairing TME homeostasis [ 6– 9]. The binding of sialylated glycans and sialic acid-binding immunoglobulin-like lectin (siglec) receptors remodeled the immunosuppressive TME through activating the tumor-promoting phenotype of tumor-associated macrophages (TAMs), repressing the activation of natural killer (NK) cells and dendritic cell-mediated antigen presentation and subsequent T-cell responses [ 10– 14]. Aberrant glycosylation also regulates the interaction between immune checkpoint molecules and their corresponding ligands by affecting their stability and/or binding affinity [15]. Thus, in-depth analysis of the role of glycosylation in the tumor immune response and how aberrant glycan structures modulate tumor-mediated immune evasion may provide new and viable strategies for potential immunotherapy.

In this review, we focus on the role of glycosylation in antitumor immunity, particularly its regulatory effects on endogenous lectins, a wide range of immune checkpoints and the ECM in the TME. We summarize the mechanisms by which aberrant glycosylation modulates the tumor immune response, ranging from influencing endogenous lectins and immune checkpoints to influencing their interactions with other molecules, as well as the function of immune cells in the TME. In addition, we also review the current glycan-based cancer immunotherapies and aim to pave the way for future investigations on targeted glycosylation to augment the efficacy of immunotherapy.

## Endogenous Lectins and Tumor Immunity

Accumulating evidence has demonstrated that endogenous lectins play vital roles in the tumor immune response by binding to specific glycan structures in the TME. Among them, siglecs and galectins are the main types of lectins and play critical roles in antitumor immunity. Here, we summarize the functions of the Siglec and galectin families in the immune response.

### Sialic acid-siglec interactions

Tumor cells are coated with large amounts of sialic acids present at the termini of glycans. These sialic acids are added to N-glycans or O-glycans through sialyltransferases in the linked forms of α2,3, α2,6, and α2,8 [16]. Siglecs, which belong to the immunoglobulin-like lectin family normally expressed on innate and adaptive immune cells, recognize and bind to sialic acid-containing glycans (sialoglycans). Based on their evolutionary relevance, siglecs are divided into two prominent subsets. Siglec-1, siglec-2 (CD22), siglec-4, and siglec-15 are conserved receptors, while others are highly variable CD33-related siglecs (siglec-3, siglec7, siglec-9, siglec-10, *etc*.) [17]. The immunoregulatory effects exerted by siglecs rely on their intracellular structural domains. Most siglecs contain immunoreceptor tyrosine-based inhibitory motifs (ITIMs) within their intracellular regions, which, in turn, recruit tyrosine phosphatases such as Src homology 2 domain-containing protein tyrosine phosphatase 1 (SHP-1) and SHP-2 to transduce inhibitory signals [18]. Therefore, the elevated levels of sialylation in cancer cells enhance their affinity for binding to siglecs, potentially dampening the immune response and facilitating immune evasion.

#### Siglec-7

Siglec-7 is an inhibitory receptor constitutively expressed in NK cells and tends to bind disialyl-T antigen with α2,3- and α2,6-linked sialic acids [ 12, 19, 20] ( Table 1). Hudak *et al*. [10] used glycopolymers end-functionalized with phospholipids to reshape the sialylation state of cancer cells, which resulted in the engagement of siglec-7 and impaired the killing function of NK cells. The protein ligands on target cells that bind to Siglec-7 include CD43 [21], CD45 [20], P-selectin glycoprotein ligand-1 (PSGL-1) [ 11, 20], MUC2 [19], and MUC5AC [19] ( Table 1). Accumulating evidence has revealed that inhibiting the interactions between Siglec-7 and its ligands enhances the anticancer activity of cytotoxic immune cells. For example, Wisnovsky and his colleagues reported that blocking the interaction between CD43 and Siglec-7 enhanced the cytotoxic activity of NK cells toward leukemia cells [21]. In multiple myeloma (MM), PSGL-1 is overexpressed and acts as a sialic acid-derived ligand for Siglec-7. Targeting the interaction between PSGL-1 and Siglec-7 could increase NK cell-mediated cytotoxicity [11]. These findings demonstrate that selective blockade of the interactions between Siglec-7 and its ligands is a promising strategy for cancer therapy.

**Table:** **Table 1** Glycans and protein ligands of siglecs in tumors

Siglec	Cell	Glycan ligand	Protein ligand	Reference
Siglec-7	NK, Mono, Macro, T	Disialyl-T, *α*2,3/6 sia	CD43, CD45, CD162/PSGL-1, MUC2, MUC5AC	[ 11, 12, 19– 21]
Siglec-9	NK, Mono, Neu, DC, TAM, T	sLex, sTn, *α*2,3 sia	ST-MUC1, MUC16, LGALS3BP	[ 19, 22– 28]
Siglec-10	TAM, T, NK,	*α*2,3/6 sia	CD24, CD52	[ 31– 33]
Siglec-15	BMDM	sTn, *α*2,3/6 sia	CD44, CD11b	[ 22, 35, 37, 38]

#### Siglec-9

Siglec-9 is another inhibitory receptor that is widely expressed in immune cells, particularly in myeloid cells, including monocytes, neutrophils and macrophages. Glycan ligands that contain sialic acid α2,3 linked to galactose are preferred ( Table 1). Several studies have demonstrated that Siglec-9 recognizes specific antigens, such as sialyl Lewis X (sLex) [ 22, 23], sialyl Tn (sTn) [ 23, 24], sialyl-T-MUC1 (ST-MUC1) [ 25, 26], and MUC16 [ 27, 28] ( Table 1). The binding of ST-MUC1 to siglec-9 activates the MEK-ERK pathway in macrophages, leading to the induction of a TAM phenotype in macrophages by increasing the expression of suppressive proteins such as indoleamine 2,3-dioxygenase (IDO), CD163, CD206, and programmed cell death ligand 1 (PD-L1) to inhibit T-cell activity [ 25, 26]. The expression of siglec-9 is frequently upregulated in glioblastoma (GBM) and is positively correlated with poor prognosis in GBM patients. Single-cell RNA sequencing and spatial transcriptomics analyses of GBM patients who did not respond to anti-PD-1 therapy revealed high and continuous expression of the *SIGLEC9* gene in TAM subpopulations [29]. Blocking siglec-9 in combination with an anti-PD-1 antibody has shown potential for enhancing immune checkpoint blockade (ICB) immunotherapy for GBM. Similar observations have been made in high-grade serous ovarian cancer (HGSOC), where high expression of siglec-9 on TAMs correlates with CD8 ^+^ T-cell exhaustion and increased expression of immune checkpoint molecules [30]. Blocking siglec-9 leads to the suppression of SHP-1 phosphorylation, resulting in TAM repolarization and the restoration of CD8 ^+^ T-cell cytotoxicity.

#### Siglec-10

Siglec-10 recognizes glycans that carry α2,3- or α2,6-linked sialic acids and is mainly expressed on the cell surfaces of TAMs, T cells and NK cells ( Table 1). Recent research has revealed that CD24, a glycosylphosphatidylinositol (GPI)-anchored protein in tumor cells, represses the innate immune response by binding to siglec-10 in a sialic acid-dependent manner [ 31, 32]. Soluble CD52 containing sialylated N-glycans is another ligand for siglec-10. It inhibits T-cell activation by dampening the phosphorylation of T-cell receptor-associated kinases, including lymphocyte-specific protein tyrosine kinase (Lck) and the zeta chain of T-cell receptor-associated protein kinase 70 (Zap70), when binding to siglec-10 [33].

#### Siglec-15

Siglec-15 is primarily expressed by myeloid cells and macrophages and plays central roles in suppressing antigen-specific T-cell proliferation, thereby contributing to immune evasion [34]. As an immunomodulator, siglec-15 remodels the TME to promote primary tumor metastasis in a manner dependent on sTn [35] and branched α2,3/6 di-sialylated biantennary glycans [22] ( Table 1). A recent study proposed that osteoclasts upregulate Siglec-15 to suppress T-cell activity, contributing to the establishment of an immunosuppressive TME and facilitating secondary metastasis in breast cancer [36]. Previous studies have indicated that CD44 on tumor cells and CD11b on T cells can act as ligands for siglec-15, suggesting that siglec-15 may interact with different ligands from various cell types, further influencing its role in tumor progression and immune regulation [ 37, 38].

**Figure 1:**
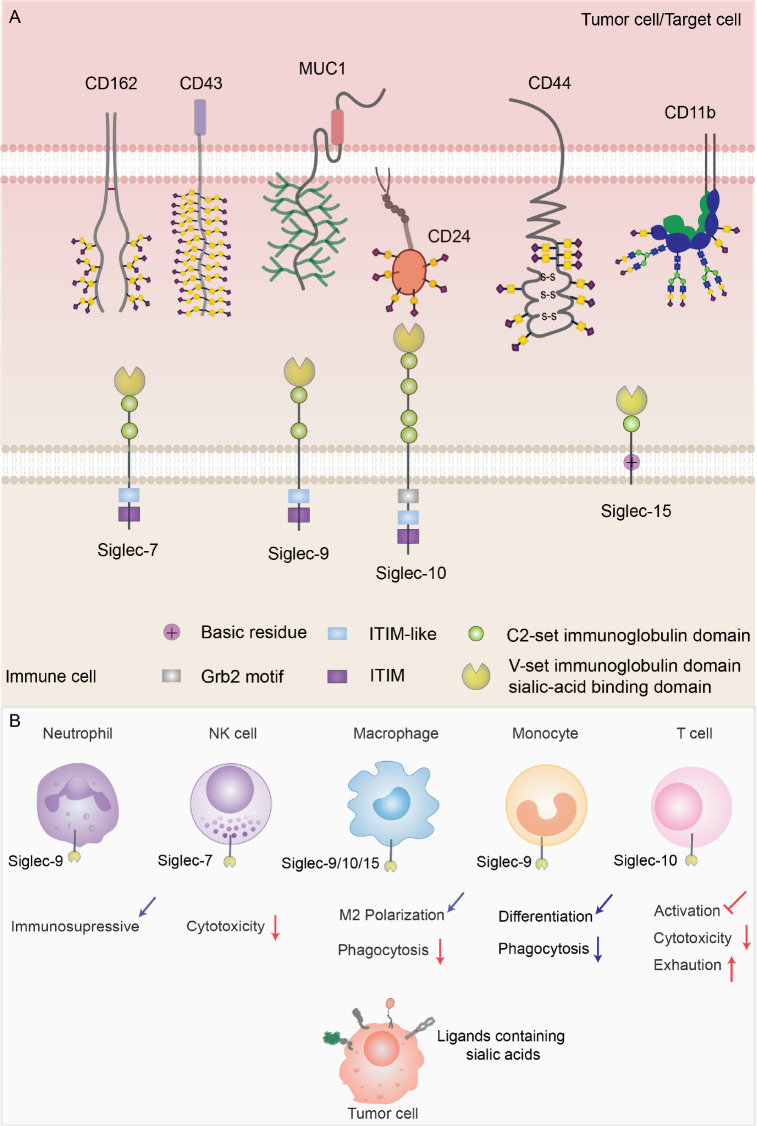
Figure 1 Ligands of siglecs and the effects on immune cells mediated by their interactions (A) Structures of siglec7/9/10/15 and their binding ligands on tumor cells or target cells. (B) Potential effects on immune cells caused by the interaction between siglec 7/9/10/15 and glycosylated ligands on tumor cells.

Thus, an increasing number of studies have shown that hypersialylation on the tumor cell surface contributes to tumor immune evasion by binding to siglecs present on immune cell surfaces. The interaction of siglecs and protein ligands with sialylation inhibits the antitumor activity of immune cells ( Figure 1). Consequently, targeting glycosylated cell-surface antigens is emerging as an attractive strategy for cancer immunotherapy.

### Glycans and galectins

Galectins, a family of β-galactoside-binding proteins, mainly recognize N-acetyllactosamine (LacNAc) epitopes on glycosylated receptors through their carbohydrate recognition domains (CRDs). Based on the structure of CRDs, galectins can be categorized into three subgroups: prototypical galectins (GAL1, GAL2, GAL5, GAL7, GAL10, GAL11, GAL13, GAL14, and GAL15), tandem repeat galectins (GAL4, GAL6, GAL8, GAL9 and GAL12), and chimeric galectins (GAL3) [39]. Prototypical galectins possess two identical CRDs, while tandem repeat galectins contain two different CRDs. Chimeric galectins have only one GAL3 member with one CRD, which is linked to a nonlectin N-terminal domain (NTD) responsible for GAL3 oligomerization [40]. Dysregulation of galectins is observed in different cancers and affects the tumor immune response through glycosylation-dependent or glycosylation-independent mechanisms. Among them, GAL1, GAL3, and GAL9, in particular, have been implicated in immune evasion. In this section, we focus on the functions of GAL1, GAL3, and GAL9 during the immune response.

#### GAL1

GAL1, a highly conserved galectin encoded by the *LGALS1* gene, is associated with diverse glycosylated ligands, such as CD7, CD43, CD45, CD69, melanoma cell adhesion molecule (MCAM/CD146), and vascular endothelial growth factor receptor (VEGFR) [ 41, 42] ( Table 2). GAL1 is abundant in the TME, where it is involved in immune regulation by supporting tumor cells in evading immune surveillance. MCAM is the main GAL1 ligand in melanoma and binds to GAL1 mostly through N-glycans [42]. Many studies have explored how GAL1 assists tumor cells in acquiring immune privilege by impacting T-cell function. Initially, GAL1 was considered a crucial factor in the apoptosis of tumor-specific effector T cells. Due to the interaction between GAL1 and its ligands with poly-N-acetyllactosamine (LacNAc), T-cell homeostasis is disrupted, which is characterized by T-cell apoptosis, inactivation, and decreased proinflammatory cytokines [43]. In addition, lung cancer cell-derived GAL1 increases the amount of CD4 ^+^CD25 ^+^FOXP3 ^+^ Tregs, an immunosuppressive T-cell subset in the TME [44]. Similarly, the depletion of Gal1 reduces the infiltration of Tregs within tumors, decreases the level of the T-cell regulatory molecule linker for the activation of T cells (LAT), and eliminates the immunosuppressive function of Tregs in breast cancer [6]. In addition to CD4 ^+^CD25 ^+^FOXP3 ^+^ Tregs, CD8 ^+^CD122 ^+^PD-1 ^+^ Tregs identified in colorectal cancer (CRC) have been associated with Gal-1. Notably, heightened GAL1 expression and elevated CD8 ^+^ Treg scores correlate with poor prognosis in CRC patients. Studies in CRC models have demonstrated that targeting GAL1 results in a decreased number of CD8 ^+^CD122 ^+^PD-1 ^+^ Tregs and reduced tumor growth [45].

**Table:** **Table 2** Glycan and protein ligands of galectins in tumors

Galectin	Glycan ligand	Protein ligand	Reference
GAL-1	Terminal LacNAc residues	CD7, CD43, CD45, CD69, VEGFR, MCAM/CD146	[ 39, 41, 42]
GAL-3	Wide range of LacNAc residues	MUC1, CD45, CD71, LAG-3, α3β1 integrin, CD98	[ 8, 39, 49, 51, 53, 58, 59]
GAL-9	Prefers poly-LacNAc repeats	TIM-3, PD-1, VISTA, CD44, Dectin-1, Dectin-2, ERMAP	[ 9, 39, 54– 57, 60– 62]

GAL1 can not only directly modulate T cells within tumors but also regulate other cells, such as endothelial cells, macrophages, and cancer-associated fibroblasts (CAFs), to form a barrier against T-cell infiltration or dysregulate T-cell function. Endothelial cells are reprogrammed by GAL1 and increase the expression of PD-L1, which leads to T-cell exclusion [7]. Hypoxia is a common feature observed in numerous tumors. This hypoxia-induced milieu triggers a reciprocal activation mechanism between GAL1 and hypoxia-inducible factor-1 (HIF), facilitated by H-Ras [46]. Under hypoxic conditions, heightened lactate production promotes the differentiation of myeloid-derived suppressor cells (MDSCs) into TAMs. Within pancreatic ductal adenocarcinoma (PDAC), over-activated pancreatic stellate cells (PSCs) secrete substantial amounts of GAL1 and interleukin-6 (IL-6) [47]. GAL1 synergizes with lactate to activate HIF, promoting the differentiation of MDSCs into TAMs [48]. On the other hand, IL-6 recruits MDSCs via the IL-6/JAK/STAT3 pathway or collaborates with lactate to induce the production of vascular endothelial growth factor (VEGF). Additionally, the increased levels of reactive oxygen species (ROS) resulted from hypoxia and lactate enhance the immunosuppressive activity of TAMs [40]. In summary, GAL1 promotes immune system evasion and induces T-cell dysfunction through the regulation of TAMs and MDSCs.

#### GAL3

In addition to CRD, GAL3 has a unique NTD because of its special ability to form pentamers. GAL3 can interact with internal or other modified LacNAc in branched N-glycans [39]. Further insights into the roles of extracellular GAL3 come from its influence on immune cells, especially T cells and macrophages. Tumor-derived GAL3 induces the apoptosis of T cells by interacting with the glycosylated receptors CD45 and CD71 [49]. The presence of GAL3 in the TME dampens immune synapse formation by hampering the mobility of T-cell receptors during T-cell activation [50]. GAL3 also binds to lymphocyte activation gene 3 (LAG-3), an inhibitory immune checkpoint, thereby suppressing CD8 ^+^ T-cell function [8]. In addition, GAL3 interacts with the glycoprotein α3β1 integrin on γδ T cells to prevent γδ T-cell proliferation [51]. In macrophages, GAL3 shows distinct expression patterns in different subtypes. Interferon-γ (IFN-γ) and lipopolysaccharide (LPS) stimulate M1 macrophages to express and release lower level of GAL3, while IL-4/IL-13-mediated M2 macrophages exhibit increased GAL3 biosynthesis and secretion [52]. Secreted GAL3 binds to the surface glycoprotein CD98 on macrophages to boost immunosuppressive M2 macrophage proliferation [53].

#### GAL9

GAL9, which belongs to the “tandem repeat galectins” family, consists of two homologous but distinct CRDs, namely, the N-terminal CRD (N-CRD) and the C-terminal CRD (C-CRD). Compared with GAL1 and GAL3, GAL9 prefers poly-LacNAc structures [39] ( Table 2). Recent research has shed light on the immunomodulatory role of GAL9 in the TME. The binding of GAL9 to the T-cell suppressive marker mucin domain-3 (TIM-3) triggers T-cell apoptosis and exhaustion [9]. In addition to TIM-3, CD44 was reported to be a potential receptor for GAL9 expressed on activated T cells. Extracellular GAL9 collaborates with transforming growth factor β (TGF-β) to interact with CD44-TGF-βRI, which promotes forkhead box protein P3 (FOXP3) expression, stabilizes FXOP3, and enhances the inhibitory function of induced Tregs (iTregs) [54]. In addition to its effects on T cells, GAL9 also plays a critical role in modulating myeloid cells. In *PTEN*-deficient GBM cells, GAL9 secretion is increased, activating TIM-3 on macrophages and downstream pathways that accelerate macrophage M2 polarization, leading to angiogenesis and supporting tumor cell growth [55]. Moreover, GAL9 interacts with innate immune receptors, namely, dendritic cell-associated C-type lectin-1 (Dectin-1) and dendritic cell-associated C-type lectin-2 (Dectin-2), which are primarily expressed on dendritic cells (DCs) and macrophages [56]. The binding of GAL9 to Dectin-1 results in macrophage differentiation and the suppression of adaptive immune responses, thereby facilitating the progression of PDAC [56]. On the other hand, GAL9 binds to Dectin-2 and ERMAP, which are expressed on Kupffer cells and cancer cells, respectively, to form a complex that signals Kupffer cells to engulf tumor cells more efficiently [57]. These findings highlight the diverse roles of GAL9 in modulating the immune response and tumor progression in the TME, providing potential targets for therapeutic interventions.

**Figure 2:**
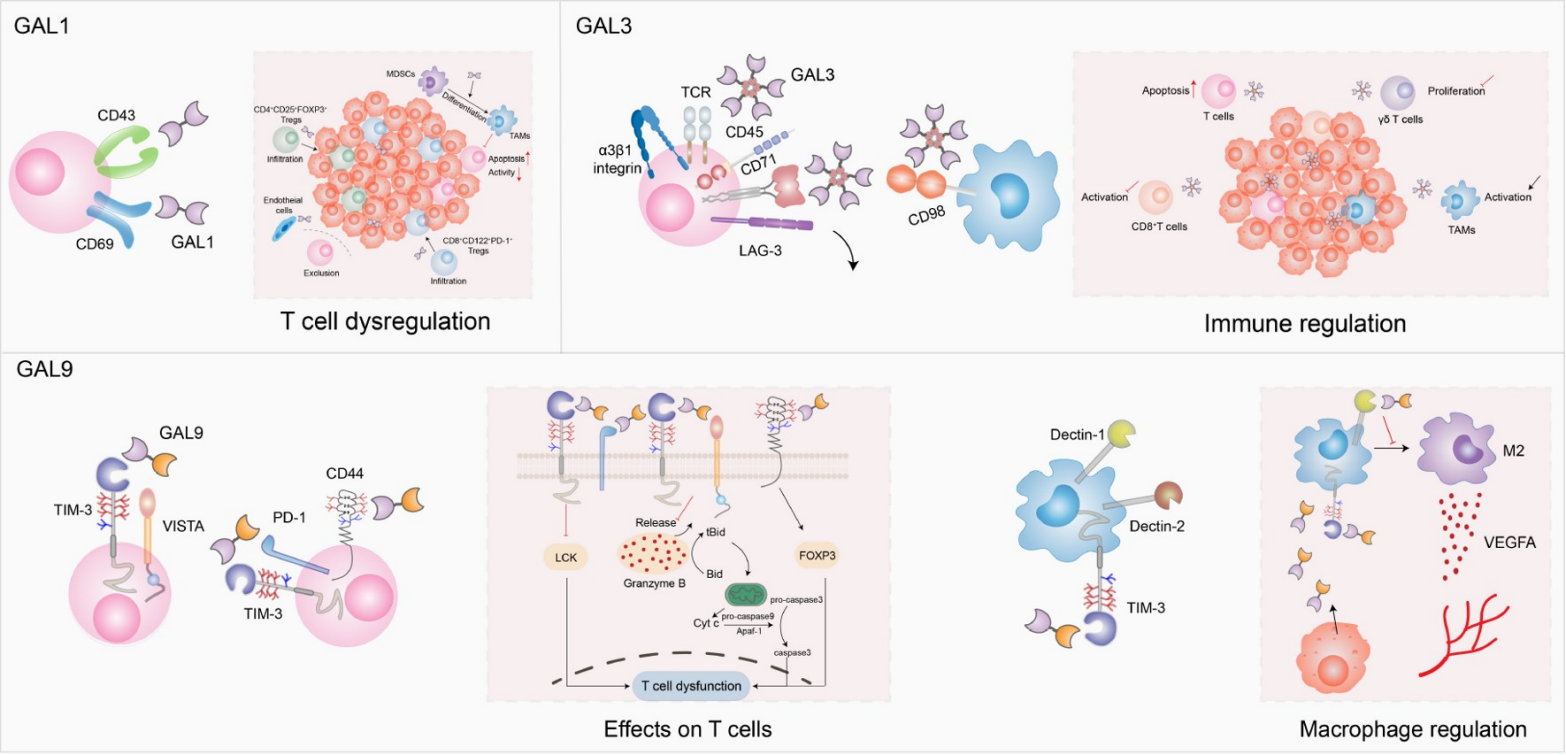
Figure 2 Interactions between galectins and their ligands regulate tumor cells and the immune system in the TME GAL1 plays a key role in T-cell regulation by influencing T-cell infiltration and function directly or indirectly. GAL3 mainly binds to cell surface receptors on T cells, such as CD45, CD71, and LAG-3, which inhibits T-cell activation and proliferation and induces apoptosis. GAL9 engages with immune checkpoint molecules, including TIM-3, VISTA, and PD-1, leading to T-cell dysregulation. GAL9 is also involved in macrophage polarization to modulate the TME.

Overall, GAL1, GAL3, and GAL9 are key players in mediating immune escape in cancer through various mechanisms, highlighting their potential as therapeutic targets in cancer immunotherapy ( Figure 2). Understanding the roles of galectins in tumor immune evasion is crucial for developing effective strategies to overcome immune suppression.

## Glycosylation of B7 Family Proteins and Tumor Immune Escape

**Figure 3:**
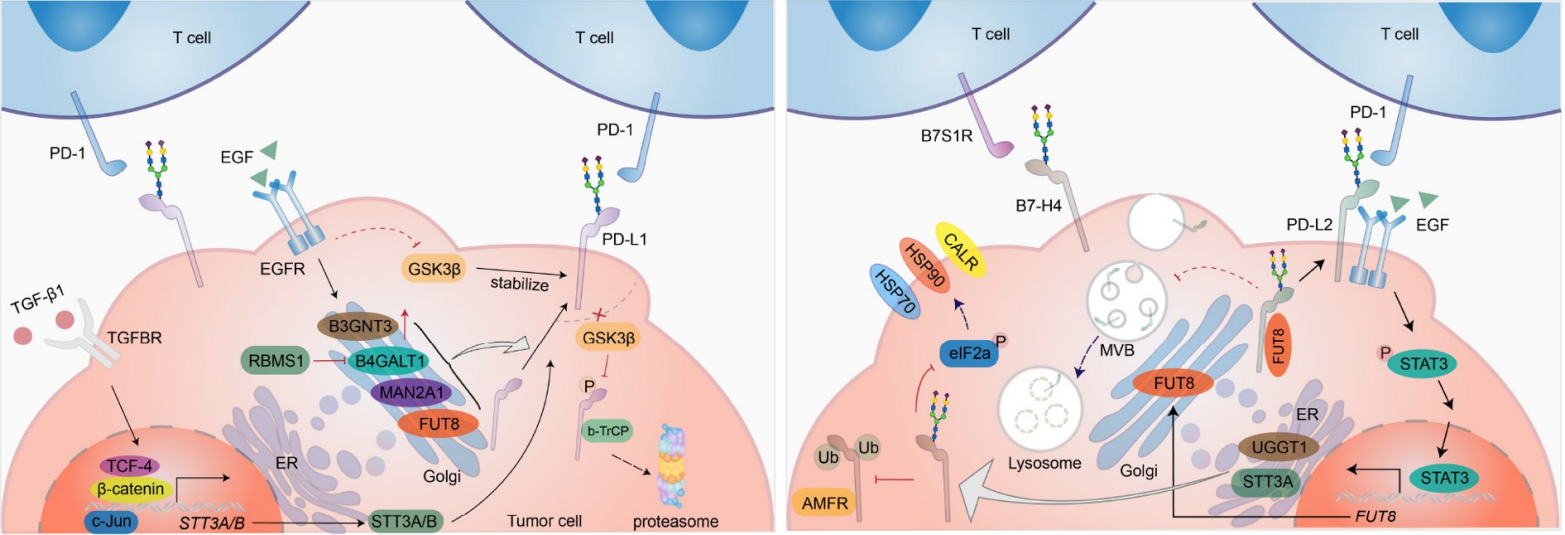
Figure 3 Regulation of B7 family glycosylation The B7 family is a group of highly N-glycosylated proteins abnormally expressed by tumor cells. Upregulated signal transduction promotes the aberrant expression of glycosyltransferases and alters the glycosylation patterns of B7 family proteins, which strengthens their stability and interaction with their ligands, thus contributing to tumor invasion.

Recent studies have shown that certain members of the B7 family, which are transmembrane proteins found on activated antigen-presenting cells (APCs), are highly expressed in tumor cells and play central roles in tumor immune evasion. To date, B7 families include ten members: B7-1 (CD80), B7-2 (CD86), B7-H1 (PD-L1 or CD274), B7-DC (CD273 or PD-L2), B7-H2 (ICOSLG or CD275), B7-H3 (CD276), B7-H4 (B7S1, B7x or VTCN1), B7-H5 (VISTA), B7-H6 and B7-H7 [63] ( Table 3). Most B7 family proteins are glycoproteins, and an increasing number of studies have highlighted the importance of how alterations in glycosylation affect proteins themselves and the TME. Here, the roles of PD-L1, PD-L2, B7-H3 and B7-H4 glycosylation in the tumor immune response are discussed ( Figure 3).

**Table:** **Table 3** Protein ligands for B7 family and the glycosyltransferases involved in B7 family regulation

B7 family	Transferase	Protein ligand	Reference
PD-L1	STT3A/STT3B, B3GNT3, B4GALT1, MAN2A1, FUT8	PD-1	[ 66, 68– 72, 84, 85]
PD-L2	FUT8	PD-1	[77]
B7-H3	FUT8	TLT-2, IL20RA, PLA2R1	[ 79, 80]
B7-H4	STT3A, UGGT1	–	[83]

### PD-L1

B7H1, also referred to as PD-L1, has emerged as a pivotal target in cancer immunotherapy. Tumor cells frequently exhibit elevated PD-L1 expression, impeding the activation of tumor-specific T cells by binding to PD-1, resulting in T-cell exhaustion and immune evasion. PD-L1 is composed of an extracellular region (including an immunoglobulin V-like (IgV) domain and an immunoglobulin C-like (IgC) domain), a transmembrane domain (TM), and an intracellular C-terminal domain [ 64, 65]. The extracellular domain (ECD) of PD-L1 contains 4 N-glycosylated sites, N35, N192, N200, and N219, which are critical for its stability and interaction with PD-1 [ 66, 67]. Compelling evidence has demonstrated that alterations in enzymatic activity during the synthesis and processing of N-glycans can lead to the erroneous formation of these glycans on PD-L1, impacting PD-L1-mediated tumor immune evasion. Epithelial–mesenchymal transition (EMT) upregulates the expression of the N-glycosyltransferase STT3 through β-catenin, leading to the stability of STT3-dependent PD-L1 N-glycosylation and contributing to cancer stem-like cell (CSC) immune evasion [68]. Mannosidase α class II member 1 (MAN2A1) is a critical enzyme that catalyzes the transformation of precursor high-mannose N-glycans to the mature complex type. Knockdown of *MAN2A1* diminishes the binding of PD-L1 to PD-1, promoting T-cell infiltration in tumors and enhancing the therapeutic efficacy of anti-PD-L1 therapy [69]. Glycogen synthase kinase 3β (GSK3β) also plays a vital role in PD-L1 glycosylation and stability. GSK3β induces PD-L1 glycosylation to block the binding of b-TrCP to PD-L1, which inhibits PD-L1 phosphorylation and proteasomal degradation of PD-L1, thereby promoting PD-L1 expression and tumor immune evasion [ 66, 70]. In addition, many glycosyltransferases and glycosidases localized in the Golgi are responsible for the processing and branching of N-glycans on PD-L1. EGFR activation increases the expression of β-1,3-N-acetylglucosaminyltransferase 3 (B3GNT3), leading to the stabilization of PD-L1, the interaction with PD-1, and the inhibition of PD-L1 internalization and degradation [71]. Another Golgi membrane protein, β-1,4-galactosyltransferase 1 (B4GALT1), also participates in regulating PD-L1 expression. B4GALT1 modulates PD-L1 N-glycosylation, preventing PD-L1 degradation. On the other hand, B4GALT1 stabilizes the TAZ protein via glycosylation, which in turn facilitates PD-L1 transcription [72]. Zhang *et al*. [73] also showed that elevating the mRNA stability of *B4GALT1* by RNA binding motif single strand interacting protein 1 (RBMS1) promotes B4GALT1 expression, thereby enhancing PD-L1 glycosylation and dampening the antitumor effect of cytotoxic T cells. Our laboratory demonstrated that O-GlcNAc modified hepatocyte growth factor-regulated tyrosine kinase substrate (HGS), a key component of the endosomal sorting machinery, and subsequently repressed its interaction with intracellular PD-L1, resulting in impaired lysosomal degradation of PD-L1 and promoting tumor immune evasion [74]. Taken together, a comprehensive understanding of the glycosylation of PD-L1 would greatly contribute to the development of new immunotherapeutic strategies.

### PD-L2

PD-L2 is a second ligand for PD-1, whose binding affinity for PD-1 is 2- to 6-fold greater than that of PD-L1 [75]. Like PD-L1, PD-L2 is a negative regulator of T-cell activation and plays a vital role in immune tolerance [76]. PD-L2 has four N-glycosylated sites, N64, N157, N163, and N189, of which N157, N163, and N189 are responsible for the PD-1 interaction rather than N64 [77]. In many solid tumors, including head and neck squamous cell carcinoma (HNSCC), PD-L2 is reportedly overexpressed and is an independent factor for poor outcomes [78]. Glycosylated PD-L2 interacts with EGFR, resulting in EGFR/STAT3 activation and decreased binding affinity of cetuximab for EGFR. STAT3 activation promotes fucosyltransferase 8 (FUT8) transcription, which is essential for PD-L2 glycosylation, as well as inhibiting PD-L2 ubiquitination and preventing PD-L2 from accessing endosomal sorting complexes required for transport (ESCRT)-mediated lysosomal degradation [77].

### B7-H3

B7-H3 is a highly glycosylated immune checkpoint molecule expressed on malignant cells and immune cells that possesses 8 N-glycosylated sites, N91, N104, N189, N215, N309, N322, N407, and N433 [79]. The abundant N-glycans on B7H3 may endow it with diverse functions and biological significance. FUT8 catalyzes core fucosylation at N-linked glycans, contributing to membrane B7H3 expression and immunosuppression in triple-negative breast cancer(TNBC). *FUT8* knockdown promotes T-cell proliferation and activation, and combined treatment with a 2-fluoro-L-fucose (2F-Fuc) inhibitor and an anti-PD-L1 antibody improves the efficacy of anti-PD-L1 immunotherapy [80]. In addition, B7H3 can interact with putative receptors on the T-cell surface, such as the triggering receptor expressed on myeloid cells (TREM)-like transcript 2 (TLT-2), interleukin-20 receptor subunit α (IL20RA), and phospholipase A2 receptor 1 (PLA2R1) [79]. However, the impact of glycosylation on the interaction of B7H3 with these ligands and the mechanisms by which it further affects T-cell function remain to be elucidated.

### B7-H4

B7-H4, also known as B7S1, B7x, and VTCN1, were identified as B7 family members in 2003 [81]. B7-H4 is normally expressed by specialized APCs but is also expressed in solid tumors. It has been reported that B7-H4 induces T cell dysfunction by interacting with B7S1R, a B7-H4 receptor on effector T cells, to increase eomesodermin (Eomes) expression, thus initiating T-cell exhaustion [82]. Extensively glycosylated B7-H4 is overexpressed in breast cancer (BC) and ovarian cancer (OV) and has 5 putative N-glycosylated sites, N112, N140, N156, N160, and N255. In TNBC, STT3 oligosaccharyltransferase complex catalytic subunit A (STT3A) and glycoprotein glycosyltransferase 1 (UGGT1) participate in B7-H4 glycosylation, which interferes with E3 ligase autocrine motility factor receptor (AMFR)-mediated B7-H4 ubiquitination. Furthermore, glycosylated B7-H4 inhibits eukaryotic translation initiation factor 2 subunit alpha (eIF2a) phosphorylation, thereby interrupting the exposure of cells to calreticulin (CALR) and the heat shock proteins, including heat shock protein 70 (HSP70) and heat shock protein 90 (HSP90), and suppressing cancer cell immunogenicity [83].

## ECM and Tumor Immunity

The ECM is an important but long-understood cellular component in tissues. Structurally, the BM is composed of the basement membrane (BM) and the stromal ECM. The BM includes laminins and collagen IV, which serve as a separating line between the layer containing endothelial and epithelial cells and the stromal ECM. The stromal ECM varies in different tissues and is characterized by alterations in components such as collagens, glycosaminoglycans (GAGs), proteoglycans, fibrillar proteins, and cytokines, which influence the molecular, physical, and mechanical features of the stromal ECM. In healthy tissues, a suitable niche is formed for the interconnectivity between adjacent cells (immune cells, fibroblasts) and the ECM. In contrast, tumor cells stimulate fibroblasts, macrophages, and other cells to be accomplices, resulting in the deposition of collagens, proteoglycans, and hyaluronic acid (HA) and ultimately the formation of an immunosuppressive environment. Here, we provide an overview of the significance and complexity of ECM components in cell-matrix interactions, TME reshaping, and tumor immune regulation.

### Collagens in tumor immune regulation

Collagens, the most abundant ECM constituents, are recognized to be critical for shaping the ECM. The synthesis of collagen initiates *COL* gene expression, followed by modifications, including proline hydroxylation and hydroxylysine glycosylation, in the rough ER (RER), which is imperative for the correct folding of procollagens. Once appropriately folded, procollagens are translocated to the outer space and become mature [86]. Collagens are predominantly expressed by CAFs in the TME. During cancer progression, the accumulation and activation of CAFs are usually observed, and the deposition of the collagen matrix is increased, providing a narrow way for immune cell infiltration [ 87, 88]. Even in the presence of high concentrations of C-X-C motif chemokine ligand 10 (CXCL10) or C-X-C motif chemokine ligand 4 (CXCL4), a T-cell chemoattractant, the infiltration of T cells in the TME is blocked due to the hypercrosslinked collagens and dense glycoproteins surrounding the tumor cells [89]. Furthermore, the collagen arrangement around tumor epithelial cell regions and vascular regions orients the migratory trajectory of T cells [90]. Collagen degradation by TAMs is also directly related to high collagen density and an immunosuppressive microenvironment. Mechanistically, TAMs ingest collagen fragments via the mannose receptor (MRC1), resulting in elevated levels of inducible nitric oxide synthase (iNOS) and reactive nitrogen species (RNS). TAM-derived RNS activate PSCs, which leads to the increased deposition of fibrillar collagens in the TME [91].

The emerging understanding is that collagens within the TME serve as ligands capable of interacting with receptors on immune cells, thus influencing tumor immunity. Various receptors participate in such interactions, including integrins, discoidin domain receptors 1 and 2 (DDR1 and DDR2), and leukocyte-associated immunoglobulin-like receptor-1 (LAIR-1) [92]. These collagen-receptor interactions lead to the modulation of tumor immunity and impact immune cell functions. For example, cancer cell-secreted collagen-I homotrimers exhibit notable resistance to cleavage by matrix metalloproteinases (MMPs) through binding to integrin α3β1 and activating the FAK-AKT-MAPK signaling pathway in PDAC cells, thereby promoting immunosuppression and tumor progression [ 93, 94]. DDR1 and DDR2, which belong to the receptor tyrosine kinase (RTK) family, are expressed by both tumor cells and some immune cells. DDR1 receptors on PDAC cells interact with collagens, inducing C-X-C motif chemokine ligand 5 (CXCL5) production, which in turn promotes the aggregation of tumor-associated neutrophils and the formation of neutrophil extracellular traps (NETs) [95]. The interaction of DDR1 with collagens in breast cancer cells leads to collagen matrix alignment and hinders immune cell infiltration [96]. In addition, LAIR-1, a coinhibitory receptor expressed on various immune cells, interacts with collagens to suppress immune cell activation through its intracellular ITIMs [ 97, 98]. Increased LAIR-1 expression and LAIR-1-dependent CD8 ^+^ T-cell exhaustion in lung cancer are induced by the interaction between integrin β1 (CD18) and collagens [99].

### Proteoglycans (PGs) in tumor immune regulation

Proteoglycans (PGs) constitute a heterogeneous array of molecules characterized by a core protein covalently linked to one or more glycosaminoglycans (GAGs). These GAGs are classified into four categories: chondroitin sulfate (CS)/dermatan sulfate (DS), keratan sulfate (KS), heparan sulfate (HS), and HA. GAGs serve as receptive sites within the TME, facilitating the binding of soluble ligands such as cytokines, chemokines, growth factors, and cell surface receptors. Delineated by their cellular localization, PGs can be divided into four groups: intracellular PGs, cell surface PGs, pericellular PGs, and extracellular PGs. Here, we summarize the pivotal roles of these PG subsets in tumor immunity.

SRGN is a unique intracellular PG found in hematopoietic cells, endothelial cells, fibroblasts, and some malignant cells that binds to inflammatory mediators to maintain them inside storage granules and secretory vesicles [100]. As exemplified by the role of SRGN in cytotoxic T lymphocytes (CTLs), it is a vector that carries granzyme B (GZMB), assisting in transporting GZMB to kill target cells [101]. SRGN can be secreted directly or by exosomes into the TME and then interacts with target ligands or receptors to regulate tumor immunity. In MM, SRGN specifically inhibits the classical and lectin pathways to protect tumor cells from attack by the complement system by binding to complement component 1q (C1q) and mannose-binding lectins (MBL) [102].

Cell surface PGs include syndecans, chondroitin sulfate proteoglycan 4 (CSPG-4), betaglycan, and glypicans, which are expressed in a variety of cancers. Syndecans consist of four members: syndecan-1, syndecan-2, syndecan-3, and syndecan-4, with HS predominantly attached to the ectodomain protein core. They also act as soluble heparan sulfate PGs (HSPGs). Syndecan-1 suppresses T-cell-mediated inflammation by binding to T-cell-specific chemokines with HS chains. A study proposed that syndecan-1 engages VEGFR2 and very late antigen-4 (VLA-4) and then causes VLA-4 S988 phosphorylation, which leads to immunosuppression [103]. Syndecan-2 expressed by tumor-associated stromal cells promotes the activation of TGF-β-mediated immunosuppressive genes, such as PD-L1 and C-X-C chemokine receptor type 4 (CXCR4) [104]. Syndecan-4 is the main HSPG expressed on the DC surface. It interacts with dendritic cell-associated heparan sulfate proteoglycan-integrin ligand (DC-HIL) via its HS chains, whose major function is to suppress T-cell activation [105]. Other cell surface PGs, such as CSPG-4, betaglycan, and glypicans, may play dual roles in modulating the TME and antitumor immunity [106].

Perlecan and arginine are multidomain HSPGs in pericellular regions that are expressed by tumor-associated stromal cells and cancer cells. The molecular states of these cells are disrupted by MMPs, sulfatases, and heparanases in the TME, which destroy the hostile stroma and reconstruct a tolerant stroma that is suitable for tumor progression and escape [107].

Extracellular PGs can be further divided into two groups: small leucine-rich PGs (biglycan, decorin, and lumican) and large extracellular PGs (versican and HA) [106]. Among small leucine-rich PGs, biglycan and decorin are usually acknowledged to play antagonistic roles in modulating the tumor immune response [108]. Biglycan facilitates the binding of TGF-β to its receptors, which initiates downstream signaling pathways and generates a protumor TME. In contrast, decorin binds to toll-like receptor (TLR) 2/4 on macrophages to promote tumor necrosis factor-α (TNF-α), C-C motif chemokine ligand 2 (CCL2), and interleukin-12 (IL-12) secretion [109]. Versican is a large matrix PG that engages with HA and linked proteins as well as cell surface proteins in the TME, such as CD44, P selectins, L selectins, and Toll-like receptor 2 (TLR2) [ 110, 111]. Tumor-derived versican dampens DC function by binding to TLR2 and forming a positive feedback loop characterized by the upregulation of IL-10/IL-6 receptors, resulting in tumor immunosuppression. HA is another important ECM component whose metabolic changes are strongly correlated with tumor-associated immune suppression. Hyaluronan synthases (HASs) catalyze the synthesis of HA, and hyaluronidase (Hyal) causes high molecular weight (HMW) HA to be fragmented into low molecular weight (LMW) HA. It appears that LMW-HA occurs in abundance in the TME because of increased Hyal expression or activity [112]. Increasing HA synthesis results in a high level of Hyal expression and ROS/NOS production, leading to HA fragmentation. On the one hand, LMW-HA bolsters the migration and proliferation of cancer cells by binding to CD44, RHAMM, and TLR2/4 [113]. On the other hand, LMW-HA formation activates fibroblasts and attracts tumor-associated neutrophils (TANs) and TAMs. Tumor-recruited Hyal2 ^+^ myeloid cells are activated in the TME to break down HA through the translocation of Hyal2 to the cell membrane, which is dependent on the CD44 signaling pathway [114]. In turn, such crosstalk promotes the differentiation of Hyal2 ^+^ myeloid cells into immunosuppressive PD-L1 ^+^ TAMs, thereby creating an immune exclusion environment [115].

Taken together, these results highlight the essential contribution of the ECM in modulating cancer-immune crosstalk. Regardless of the form, matrices containing collagens and proteoglycans link soluble factors in the TME to surface receptors on cells, which affects downstream signaling and mediates TME remodeling, thus impacting the tumor immune response. The delineated mechanisms underscore promising avenues for future therapeutic interventions aimed at targeting the ECM to modulate tumor immunity.

## Glycan-targeted Immunotherapies

In recent years, immunotherapy has led to a boom in cancer treatment research. In comparison to traditional radio- and chemotherapies, immunotherapies such as immune checkpoint blockade have made substantial progress and elevated therapeutic efficacy. Nonetheless, most patients still show low or even no response to the current existing immunotherapies. Aided by fruitful findings on how glycosylation contributes to the tumor immune response, it is promising that new strategies combined with targeting glycans will be propitious for overcoming the drawbacks of current immunotherapies. Focusing on three areas, namely, lectins, immune checkpoints and the ECM, we outline recent advances in glycosylation-targeted therapeutics ( Table 4).

**Table:** **Table 4** Agents for glycan-targeted immunotherapies.

Category	Target	Agent	Type	Status	Reference
Siglecs	Siglec7	1E8	mAb	Preclinical	[116]
Siglec9	AL009	Fc-fusion protein	Preclinical	[117]
Siglec15	NC318	mAb	Phase II	[34]
Sialic acid	E-602, E-705	Antibody-sialidases conjugates	Phase I/II, preclinical	[ 118, 119]
	Peracetyl-3Fax-Neu5Ac, 5-N-triazole substituted sialosides, 9-N-sulfonamide substituted sialosides	Sialic acid analogue	Preclinical	[ 120– 122]
Galectins	GAL1	OTX008, 6DBF7, DB16, DB21	Inhibitor	Preclinical	[ 125– 128]
	GM-CT-01	Inhibitor	Phase I/II	[129]
	F8.G7	mAb	Preclinical	[131]
GAL3	GCS-100	Inhibitor	Phase II	[130]
GAL9	P4D2	mAb	Preclinical	[133]
B7 family	PD-L1-PD-1	2-DG	Glucose analogue	Phase I	[136]
	BMS1166	Inhibitor	Preclinical	[135]
	STM108, STM418, MW11-h317, MAb059c	Glycan-targeted mAb	Preclinical	[ 70, 137– 139]
B7H3	2F-Fuc	Fucose analogue	Preclinical	[80]
B7H4	NGI-1	Inhibitor	Preclinical	[83]
ECM	HA	Hyaluronidase	Enzyme	Preclinical	[ 140, 141]
Collagen receptor	PRTH-101	mAb	Preclinical	[146]

### Lectins

#### Siglecs

Due to their expression patterns and immune-modulating functions, siglecs have emerged as appealing targets for cancer immunotherapy. One approach to block the immunosuppressive effects mediated by siglecs is through antibody-based therapies. For example, NC318, a monoclonal antibody (mAb) in a phase II clinical trial targeting siglec-15, exhibited remarkable efficacy by conferring prolonged and sustained relief to 54% of patients with refractory solid tumors [34]. Other monoclonal antibodies aimed at siglec-7/9 are undergoing preclinical development and have already demonstrated potent efficacy in reducing tumor burdens in murine models [116]. In addition, derivative antibodies, including Fc-fusion proteins, antibody-drug conjugates (ADCs), and antibody-enzyme fusions, herald a new era of therapeutics by regulating the siglec-sialic acid immune axis. AL009, an engineered Fc-fusion protein with siglec-9 ECD, competes with multiple inhibitory siglecs, thereby blocking their binding to corresponding sialic acid receptors [117]. Antibody-enzyme fusions, employing enzyme-antibody glycan-ligand editing (EAGLE) technology, combine sialidases with human mAbs, which selectively bind to tumor cells and erase overexpressed sialoglycans, thus potentiating the immune response. The drug candidate E602 is composed of two engineered sialidases and an antibody Fc fragment [118]. Although it can reverse the antitumor response both innately and adaptively in phase I clinical trials, the lack of specificity is an obstacle that limits its successful clinical translation. To overcome this drawback, anti-PD-L1 (E-705) or anti-HER2 with sialidases is designed to recognize specific tumor cells or immune cells to simultaneously remove sialic acids [ 118, 119].

Another complementary strategy involves the utilization of sialic acid analogues capable of disrupting de novo sialic acid synthesis and impeding the interaction between siglecs and sialic acids. Generally, sialic acid mimetics exhibit enhanced affinity toward the ligand-binding domain of siglecs. Modified sialic acids, such as peracetyl-3Fax-Neu5Ac, can either inhibit the production of cytidine monophosphate-N-acetyl-neuraminic acid (CMP-Neu5Ac) or sialyltransferases, leading to a reduction in sialic acid on the cell surface glycocalyx [120]. Additional small molecule inhibitors, including 5-N-triazole-substituted sialosides and 9-N-sulfonamide-substituted sialosides, play competitive roles in the interaction of most inhibitory siglecs [ 121, 122]. Beyond adopting these analogues individually, the application of liposomes, nanoparticles, and polymers as supporting materials to generate multivalent mimetics markedly enhances the avidity of siglecs and outcompetes natural ligands [ 123, 124].

#### Galectins

Within the intricate landscape of tumor immunology, the indispensability of galectins in facilitating tumor immune evasion has spurred investigations into the use of galectin inhibitors as adjuncts to immune checkpoint blockade. OTX008, an inhibitory agent of GAL1, binds to the GAL1 amphipathic β-sheet conformation rather than the CRD conformation and markedly decreases tumor cell proliferation, invasion, and angiogenesis both *in vitro* and *in vivo* [ 125– 127]. Similarly, anginex 6DBF7 and its derivatives DB16 and DB21 are noncompetitive allosteric inhibitors of Gal-1 and remain in the nascent stages of preclinical validation [128]. GM-CT-01, an α-galactomannan that targets a large surface area of GAL1, has shown no side effects on metastatic colorectal cancer patients in phase I or II clinical studies [129]. GCS-100, which is isolated from the peel and pulp of citrus fruits and modified with high pH and temperature, blocks GAL3 and has been demonstrated to potentiate antitumor effects in myeloma therapy [130]. GAL1 can also compromise anti-PD-1/PD-L1 therapy by regulating T cells or reprogramming other cells to disrupt T-cell homeostasis in the TME [ 7, 40, 43]. The highly specific neutralizing GAL1 mAb F8. G7 may reverse adverse outcomes [131]. Additionally, immune checkpoint molecules such as lymphocyte activation gene-3 (LAG-3), cytotoxic T-lymphocyte antigen 4 (CTLA-4), TIM-3, and PD-1 are binding partners for GAL3 or GAL9. Combined treatment with an anti-GAL3 or anti-GAL9 antibody (P4D2) and ICB has the potential to potentiate antitumor immunity [ 132– 134].

### B7 family

Tumor cells highly express immunosuppressive molecules, partially the B7 family, commonly modified by a unique type of glycan. Aberrant glycosylation of these molecules, which is driven by dysregulated signaling pathways in tumor cells, plays a crucial role in immune evasion and tumor progression. Based on the mechanism underlying the regulation of B7 family glycosylation, novel strategies have been investigated to address cancer therapies in recent years.

Focusing on the diverse alterations of N-glycosylation in the B7 family, agents, including small molecules, carbohydrate analogues, and glycan-targeted mAbs, have accelerated the development of immunotherapies and provided new insights into treatment. The small molecule inhibitor BMS1166 blocks the transport of PD-L1 from the ER to the Golgi apparatus and its further glycosylation, preventing PD-L1/PD-1-mediated immune suppression [135]. PD-L1 deglycosylation mediated by the glucose analogue 2-deoxy-glucose (2-DG) inhibits the PD-1/PD-L1 interaction and promotes antitumor immunity via combined therapy with gefitinib (an EGFR inhibitor) [136]. N-linked glycosylation inhibitor-1 (NGI-1), a reversible inhibitor of STT3A/B, reduces the glycosylation of B7-H4, thereby contributing to its protein stability and immunosuppressive effects in TNBC [83]. In addition, FUT8-mediated core fucosylation promotes tumor immune evasion by inhibiting the degradation of B7-H3 in TNBC. The combination of the fucose analogue 2F-Fuc [80] with an anti-PD-1 antibody synergistically promotes an antitumor immune response. Moreover, the concomitant administration of O-GlcNAc, which inhibits OSMI4, along with an anti-PD-L1 antibody further bolsters the antitumor immune response [74]. Thus, these inhibitors are instrumental in enhancing current cancer therapies in future clinical trials.

Glycan-targeted mAbs are also feasible approaches for tumor immunotherapy. STM108 is a mAb that recognizes the B3GNT3-mediated poly-LacNAc moiety modification on the N192/N200 sites of glycosylated PD-L1. When STM108 binds to PD-L1, the interaction between PD-1 and PD-L1 is blocked, and PD-L1 internalization is induced [70]. In turn, PD-1 is an N-glycosylated ligand for PD-L1 and PD-L2. The N-glycans at the N49, N58, N74 and N116 sites are important for the stability and binding of PD-L1. Compared with anti-PD-1 antibodies (nivolumab and pembrolizumab) approved by the FDA, the mAb STM418, which targets the PD-1 N58 site, exhibits greater binding affinity to PD-1, which strongly attenuates the PD-1/PD-L1 or PD-1/PD-L2 interaction, thereby enhancing antitumor efficacy [137]. Other mAbs targeting the PD-1 N58 site, such as MW11-H317 and MAb059c, also show specific binding with the PD-1 N-glycosylated antigen, resulting in a T-cell-mediated immune response and effective inhibition of tumor growth in a mouse model [ 138, 139].

### ECM

As mentioned before, the tumor-associated ECM is a barrier to immune cell infiltration. Increased HA synthesis in the TME contributes to the malignant phenotypes of many cancers. The adoption of hyaluronidase in preclinical BC and melanoma models enhances the penetration of anti-PD-L1 antibodies and cancer vaccines, thus increasing therapeutic effectiveness [ 140, 141]. Furthermore, HA-mediated ECM eradication therapy enlarges the population of antigen-specific CD8 ^+^ T cells, DCs, and macrophages in tumors and stimulates DC maturation, which induces powerful antitumor effects in cancer therapy [ 141, 142]. Blockade of ECM-binding receptors provides opportunities for cancer intervention. Several studies have demonstrated that targeting LAIR-1 in tumor models fosters immune cell activation [ 99, 143, 144]. LAIR-2 is a natural receptor that has a greater affinity for collagens [143]. Taking advantage of this mechanism, the LAIR-2 Fc fusion protein is designed to shut down the signaling pathway mediated by LAIR-1, showing notable antitumor effects [ 144, 145]. The ECD of DDR1 mediates collagen fibril alignment by binding with collagen and impeding immune cell permeability, revealing the role of DDR1-ECD in immunosuppression [96]. Recently, the humanized DDR1 antibody PRTH-101 has been proven to destroy collagen fiber alignment and increase CD8 ^+^ T-cell infiltration in tumor-bearing mice [146]. Accompanied by the accelerating focus on ECM-binding immune receptors, drugs targeting these receptors or combined therapies will continue to be developed in the future.

Tumor vaccines and chimeric antigen receptor-redirected T cells (CAR-T cells) can serve as novel approaches for targeting the intratumoral ECM. In a polyoma middle T oncoprotein mouse breast cancer model, vaccination targeting the extradomain A (EDA) of fibronectin increased macrophage infiltration and decreased tumor metastasis [147]. Fibroblast activation protein (FAP) is a key regulator of ECM remodeling and is overexpressed by CAFs. Moreover, FAP-specific CAR-T cells dramatically induce ECM degradation and suppress PDAC progression [148]. However, due to the rareness of cancer-specific ECM targets, there is still a long way to go in terms of clinical translation for tumor vaccines and CAR therapy.

## Conclusions and Prospective

Glycosylation is the most abundant and diverse form of PTM in eukaryotic cells. Sugar donors, glycosyltransferases, glycosidases, and receptors are indispensable components in the process of glycosylation, which guarantees that glycans are synthesized precisely. Fourteen glycosylation pathways, at least 173 glycosyltransferases, and other enzymes result in complex glycan structures and multiple functions of glycoproteins. The emerging field of glycobiology has shed light on the intricate interplay between glycosylation and the immune response. Aberrant glycosylation has been recognized as a hallmark of cancer. Compared with normal cells, many types of glycan alterations, including increased or incomplete glycan synthesis, hypersialylation, and elevated fucosylation, occur in tumor cells. These changes provide a more thorough profile of the tumor immune response, immune regulation, and cancer progression. The striking roles of glycans in the tumor immune response have been uncovered in the last decade and have significantly broadened the scope of cancer biology. Here, we summarize recent studies depicting how glycosylation can influence endogenous lectins, the B7 family, and the ECM, thereby remodeling the TME and protecting tumor cells from attack by the immune system. In addition, we summarize the latest advancements in tumor immunotherapy based on aberrant glycosylation, thereby providing a robust impetus for drug development and clinical interventions.

Despite substantial advancements in comprehending the pivotal role of glycosylation in the tumor immune response, research in this area still faces some challenges. For example, the complexity and diversity of glycosylation patterns pose major difficulties in elucidating the precise mechanisms by which specific glycosylation contributes to tumor progression and immune evasion. Additionally, while the interaction between glycosylation and the immune system in the tumor microenvironment has garnered considerable attention, the translation of such insights into clinical applications, such as effective glycan-based cancer immunotherapies, remains a formidable task due to the lack of specific tools for comprehensive glycan analysis. However, with the development of mass spectrometry (MS)-based glycoproteomic technology, glycan microarrays, and glycan sequencing at the single-molecule level, the precise differences in glycan structure and the resulting abnormalities in protein functions have gradually been revealed in recent years. In addition, combining multiomics data and glycomics data will accelerate the understanding of abnormal glycosylation processes in cancer development. Furthermore, it is foreseeable that the progressive acquisition and understanding of new knowledge regarding tumor glycobiology will facilitate the development of small molecule inhibitors, glycan-targeted antibodies, and other therapeutic strategies.

## Supporting information

summary_graph
