# Supplementary material for: Glycosylation in the tumor immune response: the bitter side of sweetness: Glycosylation in the tumor immune response
Source: Acta Biochim Biophys Sin (Shanghai). 2024 Jun 28;56(8):1184–98. doi: 10.3724/abbs.2024107 (PMC11399423; doi:10.3724/abbs.2024107)
Supplement: summary_graph [file summary_graph.pdf]

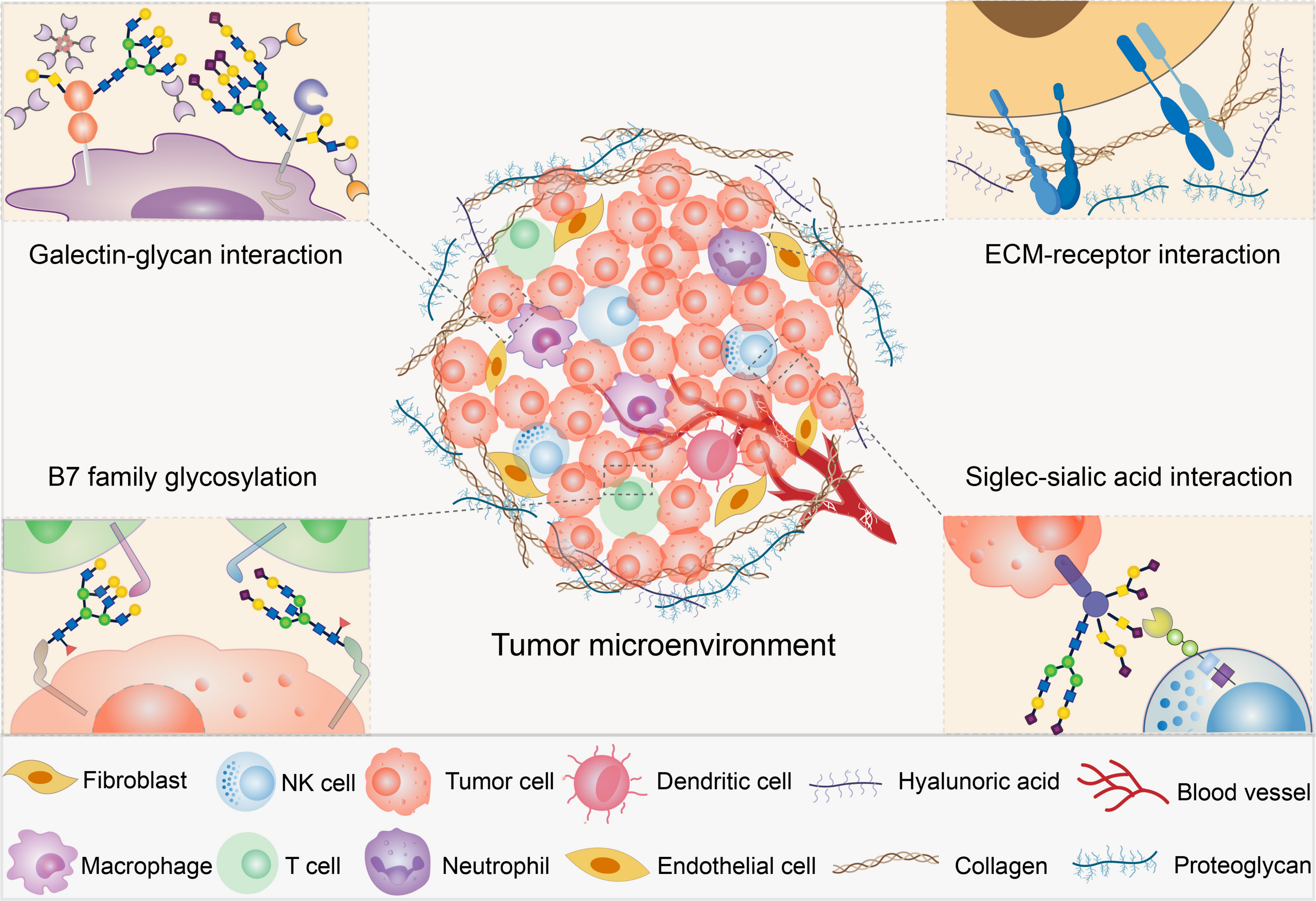
Galectin-glycan interaction
ECM-receptor interaction
B7 family glycosylation
Siglec-sialic acid interaction
Tumor microenvironment
Fibroblast
NK cell
Tumor cell
Dendritic cell
Hyalunoric acid
Blood vessel
Macrophage
T cell
Neutrophil
Endothelial cell
Collagen
Proteoglycan
